# Comparative pathology of rhesus macaque and common marmoset animal models with Middle East respiratory syndrome coronavirus

**DOI:** 10.1371/journal.pone.0172093

**Published:** 2017-02-24

**Authors:** Pin Yu, Yanfeng Xu, Wei Deng, Linlin Bao, Lan Huang, Yuhuan Xu, Yanfeng Yao, Chuan Qin

**Affiliations:** 1 Institute of Laboratory Animal Science, Chinese Academy of Medical Sciences (CAMS) and Comparative Medicine Center, Peking Union Medical College (PUMC), Key Laboratory of Human Disease Comparative Medicine, Ministry of Health, Beijing Key Laboratory for Animal Models of Emerging and Remerging Infectious Diseases, Beijing, China; 2 Institute of Laboratory Animal Science, Chinese Academy of Medical Sciences, 5 Panjiayuan Nanli, Chaoyang District, Beijing, People’s Republic of China; Deutsches Primatenzentrum GmbH - Leibniz-Institut fur Primatenforschung, GERMANY

## Abstract

Middle East respiratory syndrome (MERS), which is caused by a newly discovered coronavirus (CoV), has recently emerged. It causes severe viral pneumonia and is associated with a high fatality rate. However, the pathogenesis, comparative pathology and inflammatory cell response of rhesus macaques and common marmosets experimentally infected with MERS-CoV are unknown. We describe the histopathological, immunohistochemical, and ultrastructural findings from rhesus macaque and common marmoset animal models of MERS-CoV infection. The main histopathological findings in the lungs of rhesus macaques and common marmosets were varying degrees of pulmonary lesions, including pneumonia, pulmonary oedema, haemorrhage, degeneration and necrosis of the pneumocytes and bronchial epithelial cells, and inflammatory cell infiltration. The characteristic inflammatory cells in the lungs of rhesus macaques and common marmosets were eosinophils and neutrophils, respectively. Based on these observations, the lungs of rhesus macaques and common marmosets appeared to develop chronic and acute pneumonia, respectively. MERS-CoV antigens and viral RNA were identified in type I and II pneumocytes, alveolar macrophages and bronchial epithelial cells, and ultrastructural observations showed that viral protein was found in type II pneumocytes and inflammatory cells in both species. Correspondingly, the entry receptor DDP4 was found in type I and II pneumocytes, bronchial epithelial cells, and alveolar macrophages. The rhesus macaque and common marmoset animal models of MERS-CoV can be used as a tool to mimic the oncome of MERS-CoV infections in humans. These models can help to provide a better understanding of the pathogenic process of this virus and to develop effective medications and prophylactic treatments.

## Introduction

The Middle East respiratory syndrome coronavirus (MERS-CoV) was identified in 2012 in a cell culture taken from a patient who died of pneumonia in Saudi Arabia [1]. More men than women have become infected with this virus, and the median age of those affected is 47 years old (range: 9 months–94 years), with most of the fatalities occurring in patients over 60 years old [2,3]. The respiratory symptoms of this infection are primarily related to severe lower respiratory tract complications (e.g., dyspnoea and coughing associated with a fever) that may become fatal, while there is generally little involvement of the upper respiratory tract. A large proportion of severely ill patients require mechanical ventilation [4,5]. Complications that have been described in fatal cases include hyperkalaemia with associated ventricular tachycardia, disseminated intravascular coagulation leading to cardiac arrest, pericarditis and multi-organ failure [6].

MERS-CoV seems to be widely present in dromedary camels in the Middle East and in some parts of Africa [7,8]. Zoonotic transmission is likely to have originated from this species and is expected to continue indefinitely in these regions. The entry receptor for MERS-CoV, dipeptidyl peptidase 4 (DDP4), also named CD26, shows a high similarity in both humans and dromedary camels. Most MERS patients acquire the infection in the Middle East, which subsequently leads to limited human-to-human transmission in local groups and healthcare workers and eventually to travel-related cases outside the region, all of which can result in a mild to severe or even fatal respiratory disease [2].

Finding a suitable animal model is a major challenge for understanding the pathogenesis of MERS-CoV infection, evaluating the safety and efficacy of MERS-CoV vaccine candidates and developing therapeutic interventions. Experimental infections with MERS-CoV in rhesus macaques (*Macaca mulatta*) [9], common marmosets (*Callithrix jacchus*) [10], rabbits (*Oryctolagus cuniculus*) [11] and mice (*Mus musculus*) [12,13] have been reported in studies on the pathological changes that occur as a result of this viral infection. However, little is known about the pathological changes in the lungs of humans infected with MERS-CoV, which makes it difficult to interpret data from experimental MERS-CoV animal models. Overall, based on the known clinical aspects of MERS-CoV infection in humans, useful experimental animal models of MERS-CoV infection should exhibit a life-threatening lower respiratory tract disease.

Although there have been several studies in animal models on the pathogenic mechanisms of MERS-CoV infection, little is known about the comparative pathology and inflammatory cell response in rhesus macaques or common marmosets infected with this virus. Therefore, it is vital to study comparative pathology on the association of the MERS-CoV antigen with its receptor, DDP4, or the histopathological changes in nonhuman primate (NHP) models of MERS-CoV infection. Here, we comprehensively describe the histopathological features of the disease and the distribution of the MERS-CoV antigen and DDP4 in rhesus macaque and common marmoset models. Our findings may contribute to a better understanding of the pathogenic process of MERS-CoV infection and help in evaluating the suitability and efficacy of the animal models used in the development of effective medications and prophylactic treatments for this disease.

## Materials and methods

### Ethics statement

This research on the MERS-CoV virus was discussed among the staff members of the Department of Pathogen Biology at the Institute of Laboratory Animal Science (ILAS) of the Chinese Academy of Medical Sciences and Peking Union Medical College (PUMC). The experiments and protocols for this NHP models of MERS-CoV infection were discussed explicitly and extensively among the staff members of the Department of Pathogen Biology. These discussions were followed by consultations with biosafety officers and facility managers at the ILAS of PUMC, as well as with numerous specialists in the fields of SARS-CoV and general infectious disease research throughout China. All research procedures were approved by the ILAS Institutional Animal Care and Use Committee and the Laboratory Safety Committee (LSC). The committee recommended that the number of animals be reduced to comply with the 3R (reduction, replacement, refinement) principles. Therefore, our experiment was designed to include three rhesus macaques and three common marmosets to test their effectiveness as animal models of MERS-CoV infection. Two rhesus macaques and two common marmosets were infected with the virus and one individual of each species was left uninfected to serve as a control. The animals were planned to be euthaniazed when they were suffering from fatal respiratory symptom, impending death or 20% of body weight loss, which included fatal dyspnea and infectious shock. The approved registration number for this study is ILAS-PC-2013-004. All experiments were conducted within an animal biosafety level 3 (ABSL-3) facility, which was constructed and accredited based on National Standard GB19489 at the ILAS of PUMC, Beijing, China.

Rhesus macaques and common marmosets were housed in accordance with Chinese National standards, which are consistent with the standard set forth in the 8th edition of the NRC Guide for the Care and Use of Laboratory Animals. Because of the infectious nature of this study, NHPs were housed individually instead of the generally recommended group or social housing. Stainless steel cages measuring 0.5–0.75 m^2^ and 0.5–0.6 m depending on the weight of the individual animal, consisted of wire flooring and resting boards or perches. Rooms have natural lighting and the photoperiod is supplemented during the winter months with an artificial lighting source to provide a 12: 12 light cycle. Temperature and humidity in animal holding rooms are maintained in accordance with recommendations in the Chinese National Standards for animal care. Drinking potable water is obtained from the city of Beijing and delivered to the animals via automated watering system (AWS). The AWS is checked daily to ensure proper operation i.e., water pressure, free flowing exits and absence of leakages. Pans were cleaned daily and cages were washed every week by hand. All animals have individual cage ID cards which contain the following basic information: Study No., sex, weight, Principal Investigator's name and study protocol number. NHPs were fed a measured amount of a commercially available NHP diet (Beijing HFK Bioscience Co., Ltd) offered twice daily. Fresh fruit (apples, bananas and oranges) are supplemented on alternating days. Additional environmental enrichment consists of toys, stainless steel mirrors and heavy-duty dog chew toys (Nyla bones or similar), which are provided on a rotating basis. Toys are left inside the cages when these are transported out of the room for washing and are sanitized at this time. Damaged toys are removed from circulation. Soft background music, plants, as well as pictures and photos hung on the animal room walls are provided for relaxation. Opportunities for limited social interaction with compatible NHPs are also provided at every other cage change when cages of compatible animals are placed in close proximity to each other while avoiding direct physical contact between animals.

### Study design for nonhuman primate models of MERS-CoV infection

Two rhesus macaques, 2–3 years old, were anesthetised with ketamine hydrochloride (30 mg/kg, i.m) prior to the procedures and intratracheally inoculated with 1 mL of hCoV-EMC (6.5 ×10^7^ TCID_50_/1 mL) diluted in DMEM. One mock-infected rhesus macaque was intratracheally inoculated with tissue culture media DMEM for use as control. The rhesus macaques were observed twice daily, and clinical signs were recorded. The infected and mock-infected rhesus macaques were anesthetized with pentobarbital sodium (60 mg/kg, i.m) prior to the procedures, and while under deep anesthesia, the animals were sacrificed through femoral artery bloodletting at 3 days post-infection. Tissue specimens, including samples from lung, trachea, heart, spleen, kidney, brain, liver, and colon tissue, were collected for various pathological, virological, and immunological tests.

Two common marmosets, 2–3 years old, were anesthetised with ketamine hydrochloride (120 mg/kg, i.p) prior to the procedures and intratracheally inoculated with 1 mL of hCoV-EMC (5 ×10^6^ TCID_50_/0.5 mL) diluted in DMEM. One mock-infected common marmoset was intratracheally inoculated with tissue culture media DMEM for use as control. The common marmosets were observed twice daily, and clinical signs were recorded. The infected and mock-infected common marmosets were anesthetized with pentobarbital sodium (40 mg/kg, i.m) prior to the procedures, and while under deep anesthesia, the animals were sacrificed through femoral artery bloodletting at 3 days post-infection. Tissue specimens, including samples from lung, trachea, heart, spleen, kidney, brain, liver, and colon tissue, were collected for various pathological, virological, and immunological tests.

None of the infected animals were euthanized or died without euthanasia prior to their sacrifice at 3 days post-infection.

### Histopathological examination

The fixed samples were dehydrated and dewaxed according to conventional procedures, and 4-μm sections were prepared with a microtome. Some sections were stained with haematoxylin-eosin (HE) using routine methods. Two independent pathologists observed all slides and were blinded to the experimental design.

### Transmission electron microscopy

Lungs were fixed in glutaraldehyde and prepared for ultrastructural observations. Transmission electron microscopy was performed essentially as previously described [14].

### Immunohistochemistry (IHC)

Briefly, serial sections were dewaxed and rehydrated in graded ethanol, and a standard avidin-biotin immunoperoxidase technique was performed [15]. Table 1 lists the primary antibodies used for IHC. Optimal antibody dilutions were determined in experiments on positive control tissues. Negative control sections were prepared using the same steps as described above, but the primary antibodies were derived from an irrelevant sera.

**Table 1:** Primary antibodies used for IHC.

Antibody	Major cells expressing /Description	Source	Product number
CD68	Macrophage	abcam	ab74704
CD15	Neutrophil	abcam	ab754
MERS-CoV	Nucleoprotein of hCoV-EMC	Sino Biological Inc.	100213-RP02
DDP4	Entry receptor of MERS-CoV	abcam	ab28340
CD3	T lymphocyte	abcam	ab11089
CD4	Helper T lymphocyte	abcam	ab846
CD8	Cytotoxic T lymphocyte	abcam	ab4055
CD20	B lymphocyte	abcam	ab186523
CD57	NK cell	abcam	ab187274
CD138	Plasma cell	abcam	ab82200

### *In situ* hybridization (ISH)

Sections were dewaxed and rehydrated in a graded ethanol series. ISH was carried out using the Enhanced Sensitive ISH Detection Kit I (Boster, China) according to the manufacturer's instructions. Endogenous peroxidase activity was quenched with 0.5% hydrogen peroxide in methanol at room temperature for 30 minutes. Proteinase K digestion was performed at 37°C for 20 min. Then, pre-hybridization was performed at 37°C for 3 hours. After removing excess pre-hybridization buffer, 2 μg/ml digoxin (DIG)-modified oligo-nucleotide antisense probes (Table 2) in the hybridization solution were applied to the sections, followed by incubation at 37°C overnight. After washing the slides in 2× saline-sodium citrate (SSC), 0.5×SSC, and 0.2×SSC buffer, the sections were incubated in a blocking buffer at 37°C for 30 min. The sections were then incubated with biotinylated mouse anti-DIG at 37°C for 60 min and with streptavidin biotin peroxidase and biotinylated peroxidase for an additional 20 min, with each incubation followed by three washes in phosphate-buffered saline (PBS). The sections were treated with 3, 3-diaminobenzidine for 2 min, counterstained in haematoxylin for 5 min, dehydrated, and mounted with neutral gum. Sections for the negative controls were prepared using the same steps described above, but the antisense or sense probes were replaced with PBS at pH 7.4.

**Table 2:** DIG-modified oligo-nucleotide probe used for ISH.

Probe	Sequence (5'–3')
MERS-CoV(antisense)	5-CAGTATGTGTAGTGCGCATATAAGCA-3

## Results

### Clinical signs

Rhesus macaques were observed twice daily for clinical signs. The rectal temperature of the infected rhesus macaques increased to 40.5°C at 1–2 days post-infection, and thereafter turned to normal. The infected common marmosets showed manifest symtoms of viral infection, including severe respiratory symtoms, drastical water intake decrease and overt weight loss, and the maximum body weight loss were about 11%. None of the mock infected NHPs showed abnormal clinical signs or died during the expriment.

### Pathological findings in the rhesus macaque tissues

**Fig 1:**
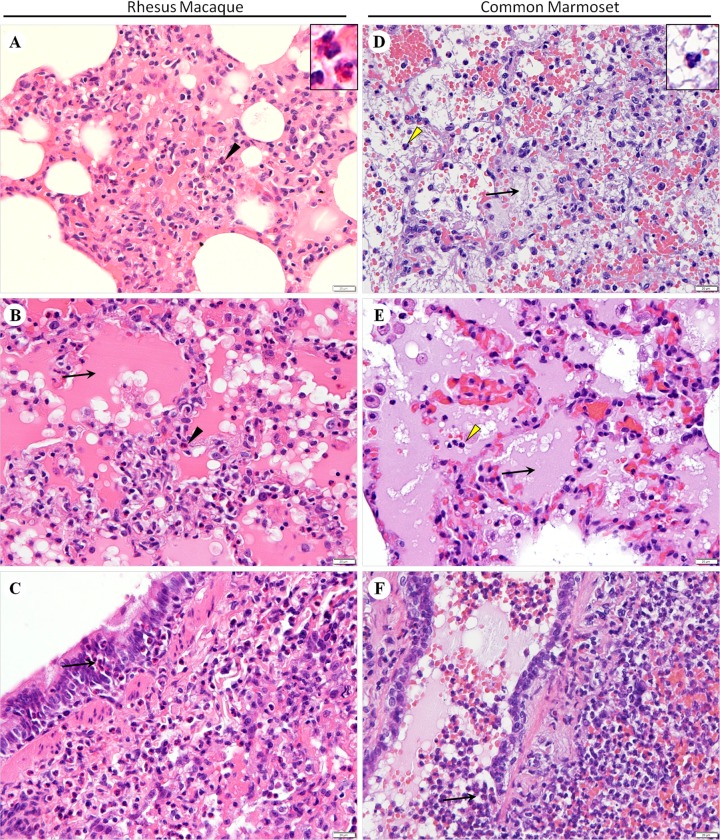
Pathological findings in the lungs of rhesus macaques and common marmosets infected with MERS-CoV. (A) Interstitial pneumonia and eosinophil (black arrowhead) infiltration in the expanded alveolar septum. (B) Pulmonary oedema (arrow) and eosinophil (black arrowhead) infiltration. (C) Necrosis of bronchial epithelial cells and eosinophil (arrow) infiltration. (D) Fibrinous exudation (arrow) and neutrophil (yellow arrowhead) infiltration in the alveoli. (E) Pulmonary oedema (arrow) and neutrophil (yellow arrowhead) infiltration. (F) Necrosis of bronchial epithelial cells and neutrophil (arrow) infiltration. Haematoxylin-eosin staining. Bars represent 20 μm.

HE stained tissues from rhesus macaques experimentally infected with MERS-CoV demonstrate that MERS-CoV induces lesions that are primarily observed in the lungs, with varying degrees of inflammation, interstitial pneumonia (Fig 1A), pulmonary oedema (Fig 1B), haemorrhaging, degeneration and necrosis of pneumocytes and bronchial epithelial cells (Fig 1C), and the infiltration of inflammatory cells. Focal interstitial pneumonia and pulmonary oedema were observed in different parts of the pulmonary lobes, as was mild haemorrhaging. The most prominent pathological effect observed in the lungs of rhesus macaques was diffuse and focal eosinophil infiltration in the thickened alveolar septum and oedematous alveolar cavities, around the bronchus, and among the necrotic bronchial epithelial cells. No significant pathological changes induced by viral infection were observed in the other organs, and no obvious pathological changes were identified in any tissues examined from the control rhesus macaque (S1A Fig).

### Pathological findings in common marmoset tissues

A histopathological analysis detected numerous lesions in the lungs of the infected marmosets. Exudative pathological changes were found, exhibiting haemorrhage, widespread pulmonary oedema and a large number of inflammatory cells. Fibrinous exudates were observed in the oedematous alveolar cavities (Fig 1D). Diffuse and focal neutrophil infiltration was found in the oedematous alveolar cavities (Fig 1E), bronchial lumen, and mildly thickened alveolar septum, around the bronchus, and among the necrotic bronchial epithelial cells (Fig 1F). No significant pathological changes induced by viral infection were observed in the other organs, and no obvious pathological changes were identified in any tissues examined from the control common marmoset (S1B Fig).

### Distribution of inflammatory cells in the lungs of rhesus macaques and common marmosets

**Fig 2:**
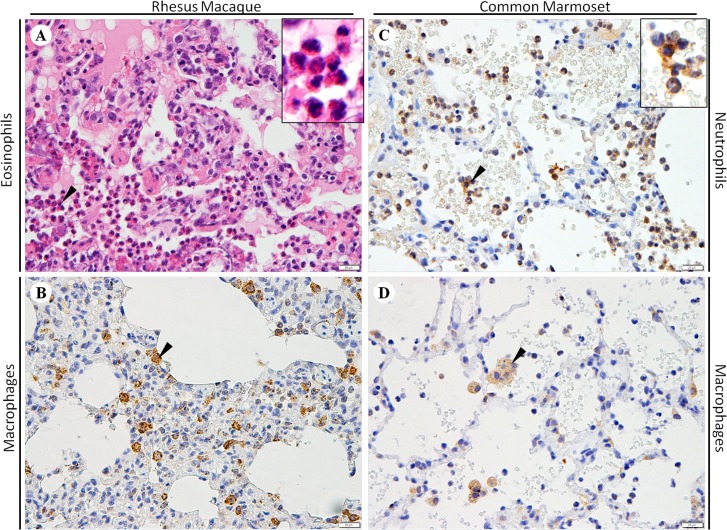
Infiltration of inflammatory cells in the lungs of rhesus macaques and common marmosets infected with MERS-CoV. (A) Numerous eosinophils (black arrowhead) infiltrated the alveolar septum. (B) Diffuse infiltration of macrophages (black arrowhead) in the expanded alveolar septum. (C) Numerous neutrophils (black arrowhead) infiltrated the oedematous alveolar cavities. (D) Diffuse infiltration of macrophages (black arrowhead). For both haematoxylin-eosin staining (A) and immunohistochemistry (B, C and D), bars represent 20 μm.

To investigate the infiltration of specific inflammatory cells, IHC was carried out to identify CD68+ macrophages, CD15+ neutrophils, CD57+ natural killer cells, CD20+ B lymphocytes, CD138+ plasma cells, and CD3+, CD4+, CD8+ T lymphocytes. In the lungs of both species, the diffuse infiltration of numerous macrophages (Fig 2B and 2D) was observed in the expanded alveolar septum and the oedematous alveolar cavities. However, in the lungs of rhesus macaques, a large number of diffusely and focally infiltrating eosinophils (Fig 2A) were found in the thickened alveolar septum and oedematous alveolar cavities, around the bronchus, and among the necrotic bronchial epithelial cells. However, in the lungs of common marmosets, numerous neutrophils (Fig 2C) infiltrated into the oedematous alveolar cavities. In both of the NHP models, other types of inflammatory cells were rarely observed.

### Distribution of MERS-CoV antigen and viral RNA in the lungs of rhesus macaques and common marmosets

**Fig 3:**
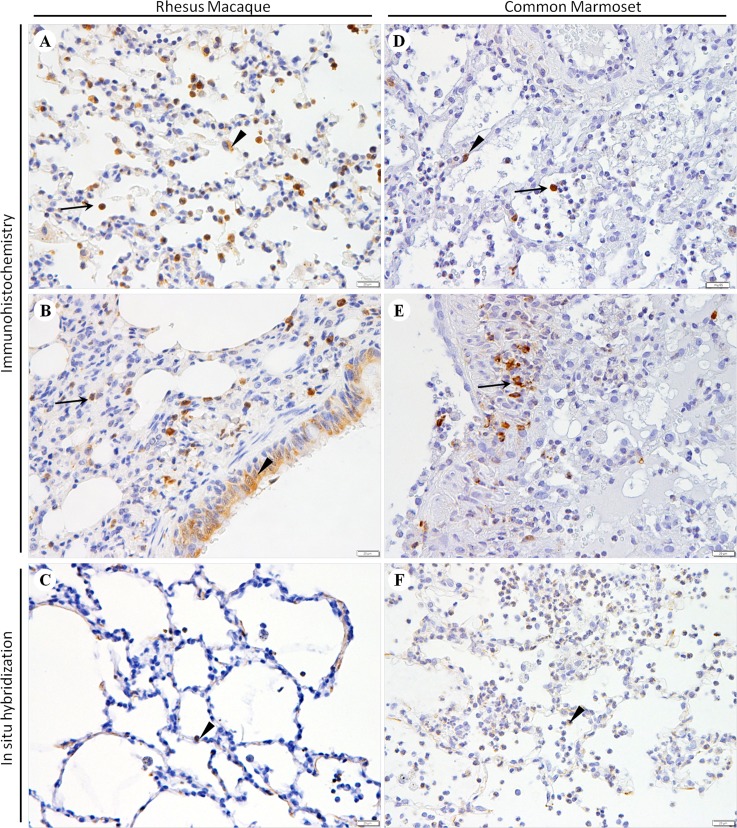
Distribution of MERS-CoV antigen and viral RNA in the lungs of rhesus macaques and common marmosets infected with MERS-CoV. (A) Viral antigen in pneumocytes (black arrowhead) and macrophages (arrow). (B) Viral antigen in pneumocytes, eosinophils (arrow) and bronchial epithelial cells (black arrowhead). (C) Viral RNA in pneumocytes (black arrowhead) and inflammatory cells. (D) Viral antigen in pneumocytes (black arrowhead) and inflammatory cells (arrow). (E) Viral antigen in the inflammatory cells (black arrowhead) around the bronchus. (F) Viral RNA in pneumocytes (black arrowhead) and inflammatory cells. For both immunohistochemistry (A, B, D and E) and *in situ* hybridization (C and F), bars represent 20 μm.

Using immunohistochemical techniques and an ISH analysis, we confirmed that MERS-CoV protein and viral RNA were distributed in the lungs of rhesus macaques and common marmosets and that they were primarily located in the pneumocytes and inflammatory cells. In the lungs of rhesus macaques, MERS-CoV antigens were extensively distributed in type I and II pneumocytes, alveolar macrophages (Fig 3A), eosinophils and bronchial epithelial cells (Fig 3B). From the microscopic characteristics, the cuboidal type II pneumocytes are located on the alveolar cavities, and smaller than macrophages. Viral RNA was also distributed in pneumocytes and inflammatory cells in the lungs of rhesus macaques (Fig 3C). In the lungs of common marmosets, a moderate level of MERS-CoV-positive antigens were detected in pneumocytes, and antigens were found more extensively in alveolar macrophages (Fig 3D), especially in the inflammatory cells around the bronchus (Fig 3E). Viral RNA was massively distributed in pneumocytes and inflammatory cells in the lungs of common marmosets (Fig 3F). No MERS-CoV-positive antigens or viral RNA was detected in the lungs of the control NHPs (data not shown). Pathological lesions and virus distribution in rhesus macaque and common marmoset animal models are summarized and shown in Table 3.

**Table 3:** Pathological lesions and virus distribution in rhesus macaque and common marmoset animal models.

**Pathology index**		**Rhesus macaque**	**Common marmoset**
**Clinical signs**		transient fever	severe respiratory symtoms, drastical water intake decrease and overt weight loss
**Pathological findings**	**Interstitial pneumonia**	focal	mild
	**Pulmonary oedema**	focal	widespread, with fibrinous exudates
	**Degeneration and necrosis of pneumocytes and bronchial epithelial cells**	focal	focal
	**Haemorrhage**	mild	diffuse in alveolar cavities
	**Infiltration of inflammatory cells**	diffuse and focal eosinophil infiltration, diffuse infiltration of numerous macrophages	diffuse and focal neutrophil infiltration, diffuse infiltration of numerous macrophages
**Virus distribution**		extensively distributed in type I and II pneumocytes, alveolar macrophages and bronchial epithelial cells	extensively distributed in alveolar macrophages, moderate distributed in pneumocytes

### Ultrastructural findings in the lungs of common marmosets

**Fig 4:**
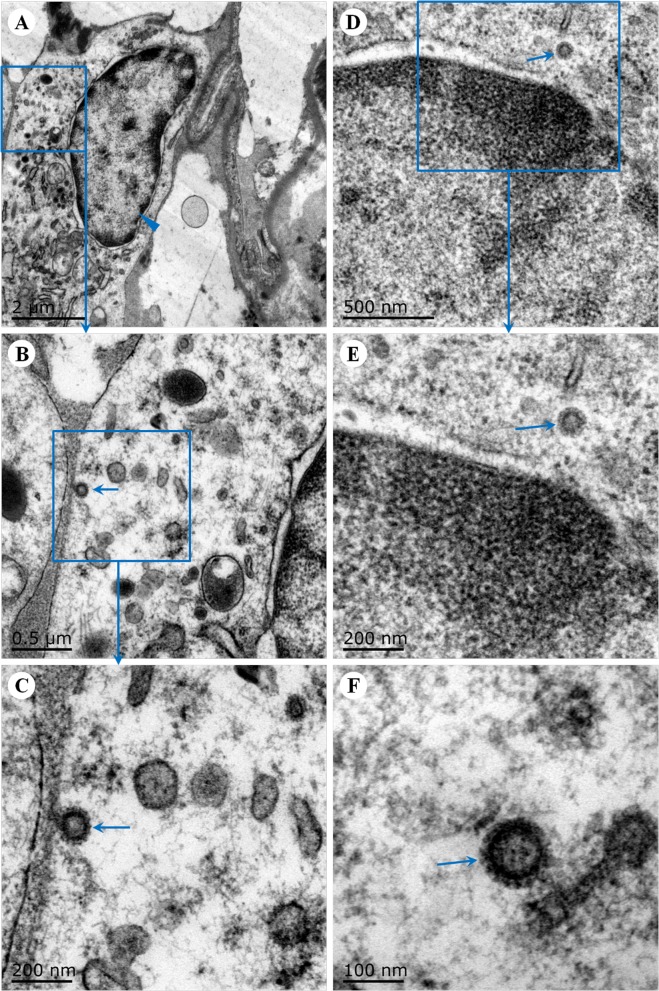
Ultrastructural pathological findings in the lungs of common marmosets infected with MERS-CoV. Virus particles (arrows, B and C) were visible in type II pneumocytes (blue arrowhead, A). Virus particles (arrow; D, E and F) were also found in the inflammatory cells in the lung (D and E). Transmission electron microscopy.

To further determine the effects of MERS-CoV infection and replication in the lungs of common marmosets, ultrastructural observations were performed on lesions in infected lung samples and on mock-infected samples. Virus particles were found in type II pneumocytes (Fig 4A–4C) and in inflammatory cells (Fig 4D–4F). Under the electron microscope, the characteristic of type II pneumocytes is lamellar bodies (S2 Fig). No viral particles were observed in the lungs of mock-infected common marmosets (data not shown).

### Expression of DDP4 in the lungs of rhesus macaques and common marmosets

**Fig 5:**
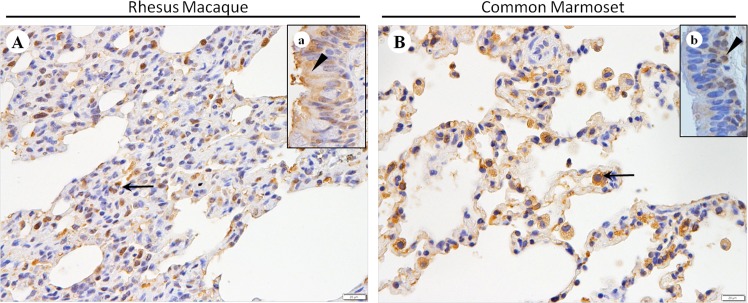
Distribution of DDP4 in the lungs of rhesus macaques and common marmosets infected with MERS-CoV. (A) DDP4 expressed in type I and II pneumocytes, inflammatory cells (arrow) and bronchial epithelial cells, primarily in ciliated cells (black arrowhead, a) and basal cells. (B) DDP4 expressed in the type I and II pneumocytes, inflammatory cells, especially macrophages (arrow), and bronchial epithelial cells, primarily ciliated cells and basal cells (black arrowhead, b). Immunohistochemistry. Bars represent 20 μm.

To elucidate the relationship between MERS-CoV and its entry receptor, DDP4, we determined the expression pattern of DDP4 in the lungs of rhesus macaques and common marmosets using immunohistochemical techniques. We found that in the lungs of rhesus macaques, DDP4 was strongly expressed in type I and II pneumocytes, bronchial epithelial cells (Fig 5A), and inflammatory cells, primarily alveolar macrophages (Fig 5A). Similarly, in the lungs of common marmosets, DDP4 was widely expressed in type I and II pneumocytes and alveolar macrophages (Fig 5B). However, DDP4 was only weakly expressed in the bronchial epithelial cells, mainly in basal and ciliated cells (Fig 5B).

## Discussion

In the present study, we analysed the histopathological features of MERS-CoV infection in rhesus macaques and common marmosets. Moreover, we compared the distribution of MERS-CoV antigens, viral RNA and DDP4 expression in the infected lungs of these species. We found that the lungs of both species exhibited varying degrees of lesions, including pneumonia, pulmonary oedema, haemorrhaging, degeneration and necrosis of the pneumocytes and bronchial epithelial cells, and inflammatory cell infiltration. Comparing the different trends in the two NHP models, it can be seen that varying degrees of inflammation, especially interstitial pneumonia, were found in the lungs of rhesus macaques, indicating mild disease and trend of chronic pneumonia; however, in the lungs of common marmosets, exudative pathological changes were found, exhibiting pulmonary oedema, inflammatory cell infiltration and fibrinous exudates, suggesting acute pneumonia. Similar to our results, previous study have also reported that rhesus macaques developed mild disease, and common marmoset exhibited potentially lethal disease[16]. However, in our study we found that the prominent inflammatory cells in the two NHP models were different, which may be the causality of process in MERS-CoV infection. In our study, the diffuse infiltration of numerous macrophages was observed in the expanded alveolar septa and oedematous alveolar cavities of both species. However, the most prominent pathological effect observed in the lungs of rhesus macaques was a diffuse and focal eosinophil infiltration in the thickened alveolar septum and oedematous alveolar cavities, around the bronchus, and among the necrotic bronchial epithelial cells. In contrast, in the lungs of common marmosets, diffuse and focal neutrophil infiltration occurred in the oedematous alveolar cavities, bronchial lumen and mildly thickened alveolar septum, around the bronchus, and among the necrotic bronchial epithelial cells. These differences in inflammatory cell infiltration suggest that inflammatory cells may function in the development of MERS-CoV infection. Additionally, it is worth noting that eosinophils and neutrophils play important roles in rhesus macaques and common marmosets, respectively, in the development of pulmonary lesions and the pathogenesis of MERS-CoV infection. In the lungs of common marmoset, pulmonary oedema exhibited much more severe than that in the lungs of rhesus macaques, which may be due to the difference of inflammatory cells in the lungs of NHP models. Similar to our results, previous studies have also reported that common marmosets infected with MERS-CoV exhibit acute bronchointerstitial pneumonia centred at the terminal bronchioles, with an influx of inflammatory cells, a thickening of alveolar septa, oedema, haemorrhaging and fibrosis in lung tissues [10]. Previous studies on rhesus macaques have shown that histological lesions induced by MERS-CoV infection were limited to the lungs, which exhibited interstitial pneumonia with a thickening of alveolar septa caused by oedema and fibrin accumulation, and small to moderate numbers of macrophages and even fewer neutrophils. In addition, alveoli tissue samples contained moderate numbers of pulmonary macrophages and neutrophils [17]. Previous studies on common marmosets infected with MERS also showed that marmosets developed a progressive severe pneumonia or interstitial lymphohistiocytic pneumonia, exhibiting hypoproteinemia consistent with high protein pulmonary effusions resulting from alveolar oedema and interstitial lymphohistiocytic pneumonia with type II pneumocyte and bronchial associated lymphoid tissue hyperplasia[18,19]. However, rhesus macaque model did not develop breathing abnormalities and showed no-to-very mild radiographic evidence of pneumonia[20]. Similar to our study, the common marmoset model of MERS-CoV infection mimics the acute and severe pathological process, yet the rhesus macaque model represents the mild infection of MERS-CoV. Thus, the NHP models meet different needs of MERS-CoV researches.

Fatal human cases of MERS-CoV infection cause upper respiratory tract illness, severe pneumonia and multiorgan failure. These cases also include exudative-phase diffuse alveolar damage with the denuding of bronchiolar epithelia, the prominent formation of hyaline membranes, alveolar fibrin deposits, type 2 pneumocyte hyperplasia, the occurrence of rare multinucleated syncytial cells, changes in alveolar septa related to oedema and increases in lymphocytes, with fewer plasma cells, neutrophils, and macrophages[21]. These findings provide evidence for the pulmonary tropism of MERS-CoV infection. The pathological features of MERS-CoV are shared by other similar respiratory illnesses, such as severe acute respiratory syndrome (SARS)-CoV [22]. Previous studies that have evaluated fatal human cases of SARS-CoV have described diffuse alveolar damage at various stages as the most characteristic feature of the disease, with SARS-CoV antigens primarily found in alveolar epithelial cells. In this study, we examined the histopathological features and the inflammatory cell response that occurs in the lungs of rhesus macaques and common marmosets experimentally infected with MERS-CoV. Our findings indicate that inflammatory cells may play a crucial role in fatal MERS-CoV infections and that the progression of this disease in our animal models may mimic the infection in humans, making these models useful for further study of the pathogenesis, prevention and treatment of MERS-CoV infections.

In this study, we analysed the distribution of MERS-CoV protein and viral RNA in the lungs of rhesus macaques and common marmosets. We found that pneumocytes and inflammatory cells were positive for MERS-CoV antigens and viral RNA. Similarly, in the lungs of both species, MERS-CoV antigens were identified in type I and II pneumocytes, alveolar macrophages and bronchial epithelial cells, viral RNA was distributed in pneumocytes and inflammatory cells, and viral protein were found in type II pneumocytes and inflammatory cells. Based on our observations, we therefore propose that MERS-CoV may proliferate and spread from the lungs through an inflammatory cell migration pathway.

It has been reported that in fatal human cases of MERS-CoV infection, viral antigens were identified in both unremarkable and necrotic bronchial submucosal glands and in pneumocytes and epithelial syncytial cells. However, macrophages showed no localization with MERS-CoV antigens in these cases [23]. Findings of pulmonary consolidation, diffuse alveolar damage, and pleural effusion are consistent with the clinical features with MERS-CoV infection [2,4,24]. In this study, we also detected MERS-CoV protein and viral RNA in type I and II pneumocytes, alveolar macrophages and bronchial epithelial cells. Therefore, our models of MERS-CoV infection using rhesus macaques and common marmosets may be suitable for use in the development of effective medications and prophylactic treatment and may serve as a tool to improve our understanding of the pathogenic process of MERS-CoV infection.

DPP4 is a single-pass type II transmembrane glycoprotein with a short N-terminal cytoplasmic tail. The structural residues comprising the receptor-binding domain have been defined via the co-crystallization of the MERS-CoV spike glycoprotein and DPP4. DPP4 is typically found in type I and II cells and alveolar macrophages. It has only rarely been detected in the surface epithelia of the nasal cavity and has also been found in a subset of mononuclear leukocytes and serous cells from submucosal glands [25]. In fatal human cases, DPP4 has been observed in scattered pneumocytes and syncytial cells, bronchiolar epithelia and endothelia, and alveolar macrophages [23,26,27]. In this study, we found that in the lungs of the NHPs infected with MERS-CoV, DDP4 was expressed in type I and II pneumocytes, bronchial epithelial cells, and inflammatory cells, primarily alveolar macrophages.

In conclusion, we established animal models of MERS-CoV infection using rhesus macaques and common marmosets, which mimic the oncome of MERS-CoV infection in humans and provide a tool that may help in better understanding the pathogenic process of this disease. They may also be suitable models for aiding in the development of effective medications and prophylactic treatments for MERS-CoV infections.

## Supporting information

S1 Fig **Lungs of control rhesus macaque (A) and common marmoset (B).** No obvious pathological changes were identified in the lungs examined from the control rhesus macaque or the control common marmoset.
(TIF)

S2 Fig Ultrastructural characteristic of type II pneumocytes in the lungs of common marmosets infected with MERS-CoV. Under the electron microscope, the characteristic of type II pneumocytes is lamellar bodies.
(TIF)

## Abbreviations

MERS: middle east respiratory syndrome
CoV: coronavirus
ISH: *in situ* hybridization
IHC: immunohistochemistry
DDP4: dipeptidyl peptidase 4
CD: cluster of differentiation
