# Supplementary figures and images for: Comparative pathology of rhesus macaque and common marmoset animal models with Middle East respiratory syndrome coronavirus
Source: PLoS One. 2017 Feb 24;12(2):e0172093. doi: 10.1371/journal.pone.0172093 (PMC5325479; doi:10.1371/journal.pone.0172093)

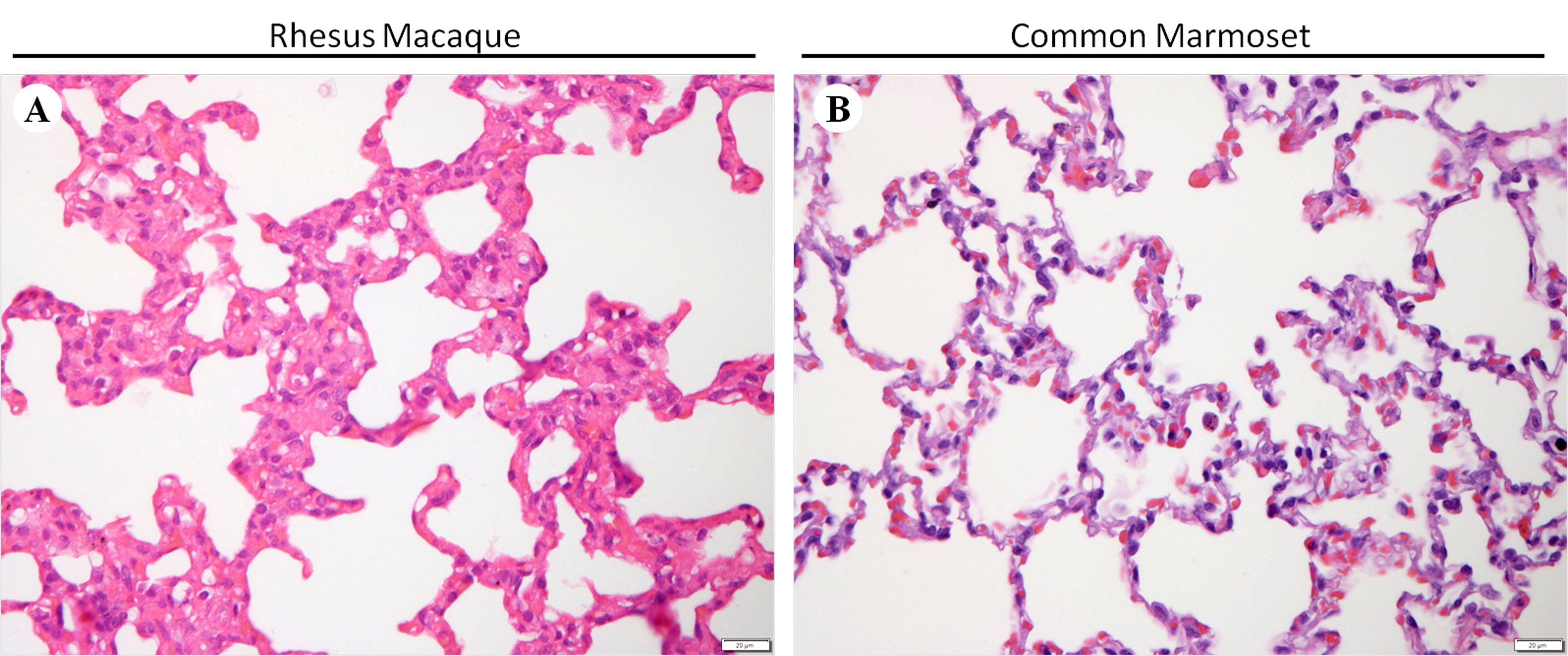

Supplement: S1 Fig — Lungs of control rhesus macaque (A) and common marmoset (B). No obvious pathological changes were identified in the lungs examined from the control rhesus macaque or the control common marmoset. (TIF) [file pone.0172093.s001.tif]

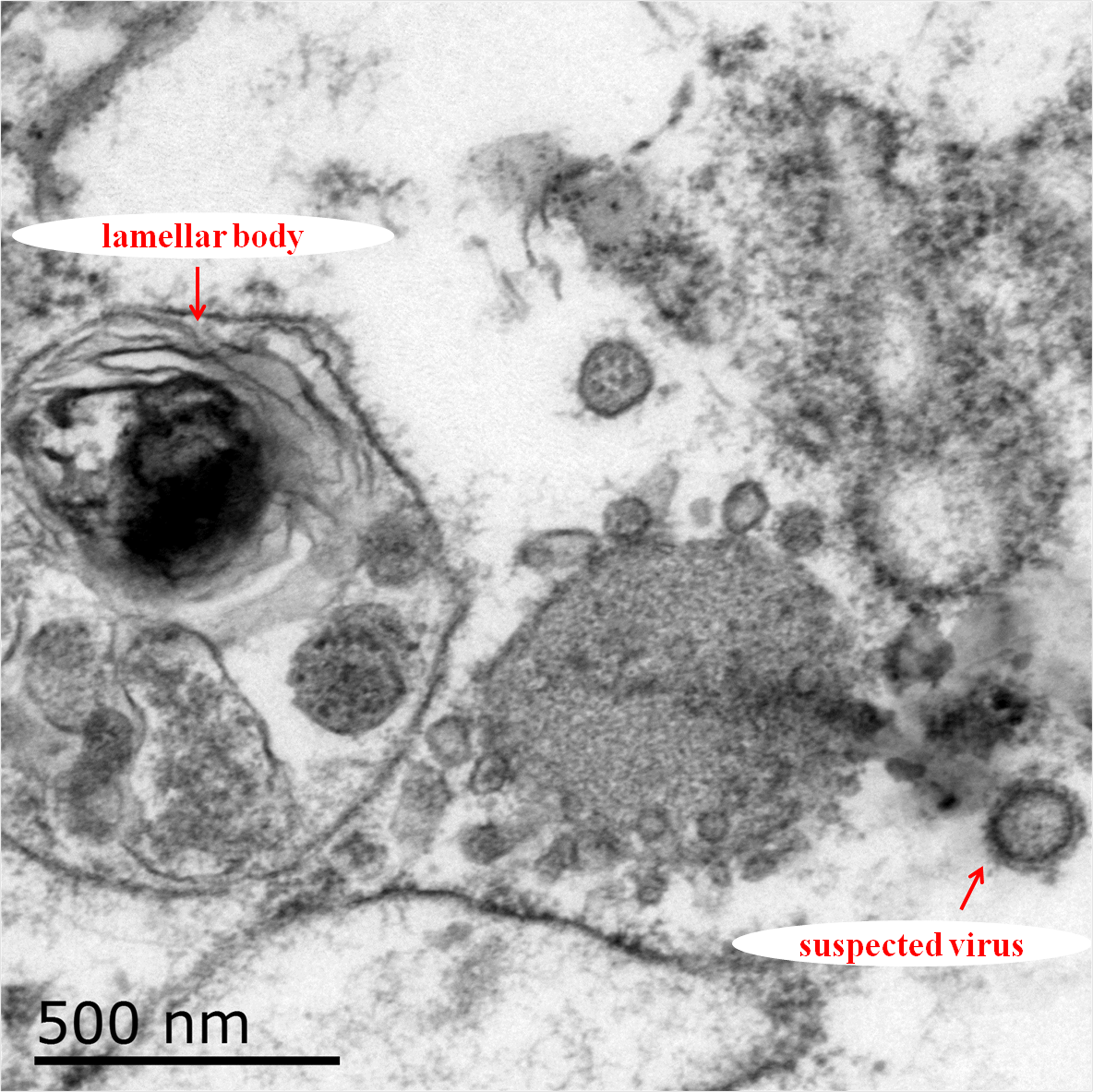

Supplement: S2 Fig — Under the electron microscope, the characteristic of type II pneumocytes is lamellar bodies. (TIF) [file pone.0172093.s002.tif]
